# Parental Knowledge, Willingness, and Attitude towards Contraceptive Usage among Their Unmarried Adolescents in Ekpoma, Edo State, Nigeria

**DOI:** 10.1155/2022/8533174

**Published:** 2022-06-23

**Authors:** Airelobhegbe Dorcas Ehiaghe, Amadou Barrow

**Affiliations:** ^1^Center of Excellence in Reproductive Health Innovation, College of Medical Sciences, University of Benin, Benin City, Nigeria; ^2^Department of Public & Environmental Health, School of Medicine & Allied Health Sciences, University of The Gambia, Kanifing, Gambia

## Abstract

**Background:**

Adolescence is a time of opportunity, vulnerability, and risk, particularly in relation to health, unsafe sexual activity, and reproductive health. Neglecting their reproductive health issues leads to a great risk of agonizing transition to parenthood, lifetime effects, and early pregnancy, which can compromise educational achievements and economic potentials. Meeting the reproductive health needs of adolescents mostly rests on the parents' shoulders. Thus, this study explored parental knowledge, willingness, and attitude towards contraceptive use among their unmarried adolescents.

**Methods:**

This was an analytical cross-sectional study design. The multistage sampling technique was used to recruit 360 parents from Ekpoma community, Edo State, Nigeria. A structured questionnaire was used to generate data for this study. Data processing and analysis were done using SPSS version 24. In order to determine association with outcome variables, chi-square (*χ*^2^) and Fisher's exact test statistics were used while statistical significance was set at *p* < 0.05.

**Results:**

The proportion of parental knowledge on contraceptive methods was at 96.9%, parental willingness for their adolescents' contraceptive uptake at 31.7%, and positive attitude at 24.2%. Associated factors for parental knowledge of contraceptives include gender (*χ*^2=^8.655, *p* = 0.003), age († = 13.377, *p* = 0.001), marital status († = 133.730, *p* < 0.001), educational level († = 103.689, *p* < 0.001), religion († = 164.592, *p* < 0.001), ethnicity († = 25.273, *p* < 0.001), and duration of marriage († = 11.365, *p* = 0.008). Factors such as educational level († = 21.220, *p* < 0.001), marital status († = 9.001, *p* = 0.022), and religion († = 6.058, *p* = 0.046) were associated with parental attitude towards contraceptives for their unmarried adolescents. Education level († = 19.905, *p* < 0.001) was associated with parental willingness for their unmarried adolescents' use of contraceptives.

**Conclusion:**

Although parents have good knowledge of contraception, this knowledge has not been passed on to their adolescents. However, few parents would encourage their adolescents to use contraceptives and advise the use of condoms if they are sexually active. Parents should be advised about contraceptive matters further to influence their adolescents' attitudes towards its usage. Establishing youth-friendly health centers will also encourage health information use and exposure.

## 1. Background

Adolescence is a time of noticeable physical, physiological, and emotional changes between childhood and adulthood [1, 2]. Adolescence is a time of opportunity, vulnerability, and risk, particularly in relation to health, unsafe sexual activity, and reproductive health [3]. The WHO defines an adolescent as 10 to 19 years old. In 2012, there were 1.6 billion people aged 12-24, 721 million of whom were adolescents, and 850 million were young adults [4]. Early adolescence is at 10-13 years, middle adolescence at 14-16 years, and late adolescence at >16 years (17-19 years) [5]. About 90% (1.8 billion) of the world's population is under 25 [6]. Sub-Saharan Africa's adolescent population will reach 23% by 2030 [7]. Most pregnancies end in miscarriage, stillbirth, or induced abortion, with 210 million women aged 15-49 becoming pregnant each year. Unsafe abortions account for 20% of all abortions globally [8]. Approximately 42 million abortions are performed annually; 20 million of these abortions are unsafe; in Africa, one-quarter of the unsafe abortions occurs between ages 15 and 19 years [9]. Adolescent pregnancy, induced abortion, and HIV/AIDS infection in Nigeria have become major problems because of adolescent sexual activity [10]. Young women who become pregnant are at greater risk of having their education, employment prospects, and mental wellbeing disrupted by their pregnancies [11]. Contraception has been shown to aid population control and reduce adolescent sexuality issues [12].

Sexual initiation during adolescence is rising in developing countries. Neglecting their reproductive health matters leads to a great risk of painful transition to parenthood, lifetime effects, and early pregnancy/motherhood, which can compromise educational achievement and economic potential [13]. Adolescent sexual activity can lead to unwanted pregnancies, which are often unplanned [14]. Girls aged 15 to 16 give birth every year, and 95 percent of these pregnancies occur in developing countries [4]. In 2020, about12 million females aged 15 to 19 and at least 777,000 under 15 years give birth annually in low- and middle-income countries [15]. Pregnancies among adolescent girls are most common in SSA, accounting for 28% of all adolescent mothers, 15% of whom are from West and Central Africa [16–18]. Pregnancies among adolescent girls are most common in Nigeria, accounting for 28% of all adolescent mothers, 15% of whom are from West and Central Africa [19]. In rural areas, 32 percent of teenagers have given birth, compared to just 10 percent in urban areas [19]. Hemorrhage and sepsis are the leading causes of hospitalization for abortion-related complications around the world [20]. Globally, an estimated 222 million women and girls do not use any form of contraception to avoid pregnancy or delay their next unwanted pregnancies [21].

Despite the eroding of traditional norms and expectations, parents still exert enormous influence on their children in many developing countries [22]. Adolescent sexual behavior is influenced by a variety of factors, including socioeconomic position, race or ethnicity, family structure, parents' educational aspirations, parental care, and life experience [23]. In families where early pregnancy or a sexually liberal attitude toward premarital sex is common, adolescents are more likely to engage in early sexual intercourse that may result in pregnancy [14]. As a result of parental closeness, sexually active adolescents are less likely to engage in prosocial development [24]. However, despite these advantages, parents avoid discussing sexual matters with their children because they fear it will encourage sexual interest and are concerned about how society will react to them [3]. Consistent contraception use helps reduce teen pregnancy and STI transmission [25]. Teenagers are discouraged from using contraceptives due to lack of access to services and parental attitudes [26].

Despite some positive sociodemographic indicators, youth in the Edo State have poor reproductive health. Nonusage of contraceptives was reported to be a result of the belief that it leads to infertility, parental influence, culture, and religion [11]. The importance of focusing on the needs of adolescents cannot be understated. There is little research on parental influence as a factor for the low uptake of contraceptives. This study looked at how parental knowledge, willingness, and attitude towards contraceptives will influence the usage of contraceptives by unmarried adolescents. Meeting the reproductive health needs of adolescents mostly rests on the parents' shoulders. Thus, this study explored parental knowledge, willingness, and attitude towards contraceptive uptake among their unmarried adolescents.

## 2. Methodology

### 2.1. Research Design

This was an analytical cross-sectional study design with a goal to systematically and factually describe parental knowledge of contraceptives, parent's attitudes towards contraceptive use among unmarried adolescents, and parent's preferred contraceptive methods for unmarried adolescents.

### 2.2. Background of Study Area

This research was conducted in Ekpoma, the administrative center of the Esan West Local Government Area in Edo State, Nigeria. The region is located between latitudes 6°431N and 6°451N and longitudes 6°601E and 6°801E [27]. Edo State is located in Nigeria's South-South geopolitical zone, bordered on the north and east by Kogi State, on the west by Ondo State, on the south by Delta State, and on the east by Anambra State [28]. Ekpoma is 364 meters above sea level. Ekpoma has 59,618 residents, ranking 4th in Edo State [29].

### 2.3. Study Population

This study was conducted in Ekpoma community among parents of adolescents of both sexes aged at least 25, and it included parents from all socioeconomic classes, religious affiliations, and civil statuses.

### 2.4. Study Variables

The dependent variable was parental knowledge, attitude, and practice on CP use among unmarried adolescents. The independent variables were sociodemographic variables (gender, education, age, educational level, religion, ethnicity, marital status, and duration of marriage), source of information on contraceptives, side effect of contraceptives, and contraceptive use among unmarried adolescents in Ekpoma Edo State.

### 2.5. Inclusion and Exclusion Criteria

Only willing parents of both sexes between the ages 25 years old and above, of any social strata or religious affiliation present at the time of data collection, were sampled for this study. Unwilling participants and those with health/mental conditions were excluded.

### 2.6. Sample Size Determination

The study's sample size was determined using Fisher's formula for a cross-sectional descriptive study. The total number of parents in the study area is above 10,000. Therefore, the sample size formula is

(1) $$\begin{array}{c} n=\frac{Z^{2}pq}{d^{2}}=\frac{\left(1.96\right)2\times 0.693\left(0.307\right)}{0.0025}=327, \end{array}$$

where *n* is the desired sample size, *z* is the standard normal deviate usually 1.96 corresponding to the 95% confidence level, *p* is the estimated proportion of the target population with a particular trait (knowledge of contraceptive prevalence for parent is 69.3%) [14], *q* = 1 − *p*, and *d* is the degree of accuracy desired set at 0.05. To compensate for nonresponse, incomplete questionnaires, etc., the sample size was increased by 10% to 360 respondents.

### 2.7. Sampling Selection Techniques

There are nine (9) quarters in Ekpoma; they are Equare, Emaduo, Ujoelen, Idumebo, Afua, Illeh, Ukpenu, Ihunmudumu, and Ujeme. A multistage sampling technique was used to select the targeted study samples. It was done in three stages: *stage 1: selection of streets*: a simple random sampling technique was used to select 2 streets out of the 10-15 streets in each quarter. A total of 18 streets were randomly selected for the study. The name of the streets in each quarter was assigned and coded in Microsoft Excel 2013 for a simple randomization function to be used to select the required number of streets. *Stage 2: selection of houses*: a systematic sampling technique sampling was used to select 10 houses out of 10-15 houses in each street. One in every two houses was selected. One hundred and eighty houses were selected for the study. *Stage 3: selection of parents*: a purposive selection technique was used to select two parents from the houses selected in stage 2. A total of 20 parents were selected per street, and 360 parents were selected for the study. However, some of the houses were with single parents who could be a father or mother. Because of this inconsistency, few other houses were added from the area.

### 2.8. Data Collection Tools

A pretested, structured questionnaire was used to interview a representative sample. The questionnaire elicits information on their sociodemographic data, parental knowledge of contraceptive provision among unmarried adolescents, parents' attitude towards contraceptive use among unmarried adolescents, and preferred contraceptive method parents think is appropriate for unmarried adolescents. A written informed consent form was given to the respondents, signed or thumb-printed. Only when approval was granted was when the questionnaire was given to the respondent, which was completed at the respondent's convenience.

### 2.9. Study Validity and Reliability

The researchers developed a questionnaire based on the research objectives. A pretest study was conducted to assess the instrument's reliability. Cronbach's alpha test was used to assess internal consistency. Following the interview, each completed questionnaire was rechecked for accuracy, completeness, and time taken to complete it. The questionnaire and the focus group guide were assessed to establish its suitability for the study by screening for any ambiguities and inaccuracies. Completed questionnaires were cross-checked by the Principal Investigator (PI) for the aspect of data quality control. Each day, the research team meets to discuss any issues that may have arisen during the research. The PI was available for any relevant clarifications based on the study.

### 2.10. Data Analysis

Data processing and analysis were done using SPSS version 24. A univariate analysis (frequencies and percentages) was carried out to elicit the predictors on the outcome variables. Cross-tabulations using two-by-two contingency tables were also used to analyze the various study objectives and provide answers to the research questions. To estimate the association between the outcome and the various risk factors, 95% confidence interval (CI) and a 0.05 significance level were calculated. For dichotomous variables, the chi-square (*χ*^2^) statistic was used. Mann–Whitney *U* test was used for ranked ordinal data that does not follow a normal distribution.

Parental attitude towards contraceptive use among unmarried adolescents was determined by 5-point Likert scales. A total of 5 different Likert items were assessed with a score of “1” to strongly disagree, “2” for disagree, “0” for neither agree nor disagree, “3” for agree, and “4” for strongly agree. With the 5-point Likert scales ranging from 0 to 4, the maximum achievable score is “20” while the minimum score is “5.” The summarized scores were computed and defined as a percentage of 50 as the benchmark. Those who scored above or equal to 50% were labeled as having a “positive attitude” and those who scored below 50% as “negative attitude.”

### 2.11. Ethical Considerations

The University of Benin's College of Medical Sciences Research Ethics Committee approved the study. The community leaders in charge of the specific areas were given letters of introduction. Parents gave written informed consent (signed or thumb-printed). There was no penalty for subjects withdrawing from the study. When analyzing the data and discussing the findings, each respondent was assured of their anonymity.

## 3. Results

A total of 360 respondents (parents) who had current or prior experience with adolescents were recruited for the study with a 100% response rate.

### 3.1. Sociodemographic Characteristics of the Study Respondents

The study respondents' mean age ± standard deviation was 38 ± 6 years, as shown in Table 1. Of the 360 respondents, 216 (60%) were females and 144 (40%) were males. Most of the respondents, 240 (66.7%), were within the age range of 25-40 years, and 120 (33.3%) were above 40 years old. The majority of 216 (60%) were married and had been in marriage for a period of 10-25years. A large proportion, 329 (91.4%), were civil servants. Two hundred and seventy-eight (77.2%) had a tertiary level of education. Christianity, 318 (88.3%), was the dominant religion.

### 3.2. Parental Knowledge of Contraceptives among Unmarried Adolescents

Three hundred and forty-nine (96.9%) of the respondents had knowledge about contraceptive methods, out of which 313 (89.7%) of the respondents knew that contraceptives are used for preventing pregnancy. For 293 (84.0%) of the respondents, their sources of information on contraceptive methods were from health personnel in the health facilities. Most of the respondents, 323 (89.7%), have heard about condom use. Three hundred and one (83.6%) reported that contraceptives have side effects. Weight gain was the most reported by 197 (60.1%) of the respondents, as shown in Table 2. Three hundred and six (85.0%) preferred only married women to use contraceptives, and slightly more than half of the respondents, 214 (59.4%), reported that contraceptives are not beneficial to unmarried adolescents.

### 3.3. Parental Attitude towards Contraceptive Usage among Unmarried Adolescents

Table 3 shows the parental attitude towards contraceptive use among unmarried adolescents in which 179 (49.7%) supported talking about contraceptives to their unmarried adolescent; an almost similar figure of respondents, 155 (43.1%), also feel free talking about contraceptives with their unmarried adolescent. One hundred and fifty-three (42.5%) did not agree to only males using contraceptives. However, 125 (34.7%) did not support adolescents' use of contraceptives because it promotes promiscuity, while 109 (30.3%) were not in support of children using contraceptives because of culture and religion, as shown in Table 3.

**Figure 1:**
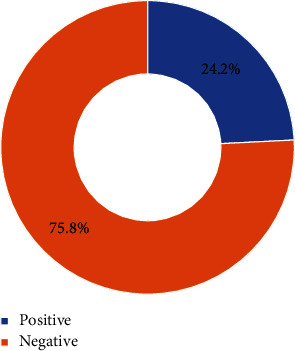
Showing parental attitude towards contraceptive use among unmarried adolescents.

As shown in Figure 1, 87 (24.2%) had a positive attitude while 273 (75.8%) had a negative attitude towards contraceptive use by unmarried adolescents.

### 3.4. Parental Willingness on Unmarried Adolescent Contraceptive Uptake

Regarding parental willingness towards contraceptive usage for unmarried adolescents, 114 (31.7%) reported encouraging them to use contraceptives, as shown in Table 4. One hundred and twenty-nine (90.2%) would prefer condoms for unmarried adolescents, 38 (19.6%) would buy contraceptives for unmarried adolescents, and 232 (64.4%) would refer their sexually active adolescent to health facilities. Sixty-seven (46.9%) would allow their unmarried adolescent to go and buy contraceptives, while 254 (70.6%) would rebuke their adolescents if they see contraceptives with him/her.

### 3.5. Sociodemographic Factors Associated with Parental Knowledge of Contraceptives

Table 5 shows the analysis of the sociodemographic factors related to parental knowledge of contraceptives. Parental gender (*χ*^2^ = 8.655, *p* = 0.003), age († = 13.377, *p* = 0.001), marital status († = 133.730, *p* < 0.001), educational level († = 103.689, *p* < 0.001), religion († = 164.592, *p* < 0.001), ethnicity († = 25.273, *p* < 0.001), and duration of marriage († = 11.365, *p* = 0.008) were found to be significantly associated with parental knowledge of contraceptives.

### 3.6. Sociodemographic Factors Associated with Parental Attitude towards Contraceptive Usage

Table 6 shows that 82 (29.5%) respondents who had a tertiary level of education had a positive attitude towards contraceptives; this significantly influences parental attitude towards contraceptive use among unmarried adolescents († = 21.220, *p* < 0.001). Other factors include marital status († = 9.001,*p* = 0.022), educational level († = 21.220), and religion († = 6.058,*p* = 0.046).

### 3.7. Sociodemographic Factors Associated with Parental Willingness for Contraceptive Usage

Table 7 shows that 85 (30.7%) respondents who had tertiary education would encourage their unmarried adolescents to use contraceptives; this was statistically significant († = 19.905, *p* = 0.001).

## 4. Discussion

This study assessed parental knowledge and attitude towards contraceptive use among unmarried adolescents in Ekpoma Edo State. The mean age was 38, consistent with studies in the Gambia and River State [30–32]. A greater proportion of study respondents attained tertiary education, which might be as a result of the availability and proximity of the state university in the study area. This is also in line with the study done at Southwest Nigeria [33] against the study at Ekpoma where the author looked at knowledge and attitude towards contraceptives among maternal women [34]. Studies show that education and contraception are directly associated [11, 34]. Parental knowledge of contraceptives and their usage is influenced by their level of education [35].

The occupation was found to be statistically significant to the parental knowledge of contraceptives. Studies have shown that occupation influences parents' decision-making and give them a higher sense of freedom in making decisions in the family [36]. From this study, many respondents were civil servants; this might be a result of the state university and other government parastatals in the area. According to the population distribution on religion, Christianity has the highest percentage, which corresponds with the study by Osemwenkha [37]. Religion is one of the major determinants of an individual's social and personal behavior within the family [38]. This study found a significant link between religion and contraceptive knowledge. Most of the respondents have heard of contraceptives, and this was also found in some other studies such as by Nansseu et al. in Cameroon [39], Barrow et al. in the Gambia [32, 40], and Adefalu et al. in Nigeria [41]. From this study, the source of information was majorly from health facilities, contradicting the study in the southern part of Nigeria, where most of their source of information were friends and relatives [41]. However, it is essential to note that health care providers can help women learn more about family planning and the various contraceptive methods available and their benefits and drawbacks. Almost all the respondents have good knowledge of contraceptives, with each respondent knowing at least one of the contraceptive methods. This was also observed in a study in Ethiopia that most respondents had a good understanding of contraceptive methods [40].

The respondents reported the prevention of unwanted pregnancy and prevention of STI as the benefit of contraceptives, according to the *Family Planning: a Global Handbook for Providers* [42]. Some of the side effects reported by the respondent were major weight gain and condom burst/spillage, while others indicated infertility. Concern over side effects is critical, as an inability to endure them can lead to nonadherence, failure of contraceptive methods, and even discontinuation of contraceptive use. People who stop using contraception due to side effects often do not use substitutes or choose less reliable options, resulting in over a million unplanned pregnancies [43]. In this study, more than half of the respondents believe that unmarried adolescents should not use contraceptives because they are too young to use them, and contraceptives are meant for married women. Parents' ignorance about sexual reproductive health may lead to misconceptions passed on to their children, influencing them to make poor sexual choices [22, 44]. The study found that despite good knowledge of contraceptives, more than half of respondents had a negative attitude toward unmarried adolescent contraception use. Unmarried adolescents are not expected to initiate sexual activities until they are married, so encouraging adolescents to obtain contraceptives is inappropriate. The Akoko-Edo and Estako West Local Governments' study corroborated these conclusions. Teenagers accessing reproductive health services were viewed negatively by most respondents in Edo [45].

Unlike the Mturi study in Lesotho, where some respondents reported being shy and embarrassed to discuss these issues, others said adolescents are still too young for this type of conversation, and others were restricted due to the cultural taboo of discussing sexual issues with unmarried adolescents [46]. The only sociodemographic factor found to be statistically significant in this study was education. This is in line with Kirby et al., who found that after undergoing training on adolescent sexuality, majority of the parents will allow their adolescent to use contraceptives, while 18% will still not [47]. This study also showed that most of the respondents do not believe in talking about contraceptives to adolescents because it promotes promiscuity; this is different from some studies showing that telling about contraception does not increase adolescent sexual life [18, 47, 48]. Majority of the respondents in this study will not encourage their unmarried adolescent to use contraceptives because they feel contraceptives are made for married people, not for the unmarried.

## 5. Conclusion

The majority of respondents knew that contraception is used to prevent pregnancy and STDs. Although parents have good knowledge of contraception, this knowledge has not been passed on to their adolescents. Most respondents knew that teen contraception prevents STDs and pregnancy. The study found that respondents will not discuss contraception with their adolescents due to the misperception that adolescents are too young to use contraception and fear side effects. However, few parents would encourage their adolescents to use contraceptives and advise the use of condoms if they are sexually active. Some parents would still react negatively if they were found using contraceptives or even prefer sending their adolescents to health facilities to get more information on their sexual lives. To dispel myths about adolescent contraception, both parents must be educated. Parents should be advised further about contraceptive matters to influence their adolescents' attitudes towards its usage. Establishing youth-friendly health centers will also encourage health information use and exposure.

## Figures and Tables

**Table 1:** Sociodemographic characteristics of study respondents.

Variables	Frequency (*n*)	Percentage (%)
Age of respondents in years		
25–40	313	86.9
41–56	40	11.1
≥57	7	1.9
*Mean (*±*standard deviation): 38 (±6)*		
Gender		
Male	78	21.7
Female	282	78.3
Marital status		
Married	297	82.5
Unmarried/single	42	11.7
Divorced	13	3.6
Widow	8	2.2
Occupation		
Civil servant	329	91.4
Artisan	26	7.2
Farmer	5	1.4
Educational level		
Primary	32	8.9
Secondary	3	0.8
Tertiary	278	77.2
Others^∗^	47	13.1
Religion		
Christianity	318	88.3
Muslims	12	3.3
Traditional religion	30	8.3
Ethnicity		
Esan	344	95.6
Benin	9	2.5
Others^∗∗^	7	1.9
Duration of marriage (*n* = 318)		
10-25	304	95.6
26-41	12	3.8
≥42	2	0.6
*Mean (standard deviation): 13.8 (±4.5)*		

Tertiary (diploma (4.2%), nursing (5.0%), polytechnic (15.5%), BSc (38.5%), MSc (10.8%), PhD (3.1%)). ^∗^Others (vocational studies (4.0%), never attended school (8.1%)). ^∗∗^Others (Igbo (0.5%), Auchi (0.5%), Hausa (0.14%), Etsako (0.4%), and Yoruba (0.36%)).

**Table 2:** Parental knowledge of contraceptives.

Variable	Frequency (*n*)	Percentages (%)
Know about contraceptive methods		
Yes	349	96.9
No	11	3.1
Meaning of contraceptives∗		
Method of preventing pregnancy	313	89.7
Method of preventing STI	124	35.5
Method of increasing sexual pleasure	22	6.3
Method of terminating pregnancy	35	10.0
Method to control family size	246	70.5
Method of abortion	4	1.2
Source of information on contraceptives^∗^		
Health personnel/health facility	293	84.0
Friends/relatives	149	42.7
Printed media/postal & hand bills	112	32.1
Electronic media	103	29.5
Husband/partner	21	6.0
Seminar/training	60	17.2
Church	28	8.0
Mosque	1	0.3
Internet	1	0.3
Knowledge of contraceptives		
Yes	323	89.7
No	11	3.1
Knowledge on types of contraceptives^∗^		
Condom	306	94.7
Diaphragm	49	15.3
Intrauterine device	129	40.3
Emergency contraceptives	199	61.6
Progesterone only	179	55.4
Combined pills	179	55.4
Depo-Provera	161	49.8
Noristerat	145	44.9
Norigynon	139	43.0
Breastfeeding	147	45.5
Traditional method (herbs 21.4%)	69	21.4
Coitus interruptus (withdrawal)	174	53.9
Rhythm method	124	38.4
Implanon	100	31.0
Female sterilization	98	30.3
Male sterilization	72	22.3
Benefits of contraceptives^∗^		
Increase sexual pleasure	54	15.1
Prevent unwanted pregnancy	348	96.7
Prevent STIs	186	51.7
To prevent abortion	11	3.1
Are contraceptives harmful or have a side effect		
Yes	301	83.6
No	32	8.9
I do not know	27	7.5
Side effects of contraceptives^∗^		
Weight gain	197	60.1
Weight loss	102	31.1
Condom burst/spillage	154	47.0
Absence of menstruation	131	39.9
Heavy menstruation	120	36.6
Irregular menstruation	129	39.3
Infertility	29	8.8
Do not know any	30	9.1
Who should use contraceptives^∗^		
Married	306	85.0
Single	138	38.3
Male only	9	2.5
Female only	58	16.1
Both male & female	203	56.4
Consequences of unprotected sex^∗^		
Unintended pregnancy	320	88.9
Sexually transmitted infection	316	87.8
HIV/AIDS	297	82.5
Infertility	44	12.2
None of the above	5	1.4
All of the above	27	7.5

^∗^Multiple responses.

**Table 3:** Parental attitude toward contraceptive use among unmarried adolescents.

Variable	*n*	Strongly disagree *n*(%)	Disagree *n*(%)	Undecided *n*(%)	Agree *n*(%)	Strongly agree *n*(%)
I support talking about contraceptives to my unmarried adolescent/child.	360	31(8.6)	50(13.9)	9(2.5)	91(25.3)	179(49.7)
I feel free to talk about contraceptives with my unmarried adolescent/child.	360	28(7.8)	52(14.4)	14(3.9)	111(30.8)	155(43.1)
Only males should use contraceptives in my own opinion.	360	148(41.1)	153(42.5)	35(9.7)	14(3.9)	10(2.8)
I do not want my adolescent to use contraceptives because it encourages promiscuity.	360	63(17.5)	72(20.0)	45(12.5)	125(34.7)	55(15.3)
I do not support my children's usage because is against my culture and religion.	360	70(19.4)	87(24.2)	41(11.4)	109(30.3)	53(14.7)

**Table 4:** Parental willingness on their unmarried adolescent's contraceptive uptake.

Variables	Frequency (*n*)	Percentage (%)
Willingness to encourage your unmarried adolescent to use contraceptives		
Yes	114	31.7
No	217	60.3
Maybe	29	8.1
Which contraceptives method will you encourage^∗^		
Condom	129	90.2
Intrauterine device	6	4.2
Diaphragm	13	9.1
Emergency contraceptives	36	25.2
Progesterone only	28	19.6
Combined pills	28	19.6
Coitus interruptus	39	27.3
Rhythm method	31	21.7
Traditional methods	8	5.6
Implanon	12	8.4
Jadelle	10	7.0
Depo-Provera	11	7.7
Noristerat	12	8.4
Norigynon	12	8.4
None	102	71.3
Will you buy contraceptives for unmarried adolescent		
Yes	28	19.6
No	102	71.3
Maybe	13	9.1
Will you refer your sexually active adolescent to health facility to obtain knowledge of contraceptives		
Yes	232	64.4
No	90	25.0
Maybe	38	10.6
Will you allow your adolescent to go and buy contraceptives		
Yes	67	46.9
No	76	53.1
Will you rebuke your adolescent if you see him/her with contraceptives		
Yes	254	70.6
No	106	29.4

^∗^Multiple responses.

**Table 5:** Sociodemographic factors associated with parental knowledge of contraceptives.

Sociodemographic variables	Parental knowledge of contraceptives			Test statistics	*p* value
	Yes *n*(%)	No *n*(%)	Total *n*(%)		
Gender					0.003^∗^
Male	63(80.8)	15(19.2)	78(100.0)	*χ* ^2=^8.655	
Female	260(92.2)	22(7.8)	282(100.0)		
Age of respondent					0.001^∗^
25-40	288(92.0)	25(8.0)	313(100.0)	† = 13.377	
41-56	31(77.5)	9(22.5)	40(100.0)		
≥57	4(57.1)	3(42.9)	7(100.0)		
Marital status					<0.001^∗^
Married	293(98.7)	4(1.3)	297(100.0)	† = 133.730	
Unmarried/single	11(26.2)	31(73.8)	42(100.0)		
Divorced	12(92.3)	1(7.7)	13(100.0)		
Widow	7(87.5)	1(12.5)	8(100.0)		
Occupation					0.239
Civil servant	297(90.3)	32(9.7)	329(100.0)	† = 2.489	
Artisan	21(80.8)	5(19.2)	26(100.0)		
Farmer	5(100.0)	0(0.0)	5(100.0)		
Education level					<0.001^∗^
Primary	7(21.9)	25(78.1)	32(100.0)	† = 103.689	
Secondary	3(100.0)	0(0.0)	3(100.0)		
Tertiary	270(97.1)	8(2.9)	278(100.0)		
Others^∗^	43(91.5)	4(8.5)	47(100.0)		
Religion					<0.001^∗^
Christianity	315(99.1)	3(0.8)	318(100.0)	† = 164.592	
Muslim	4(33.3)	8(66.7)	12(100.0)		
Traditional religion	4(13.3)	26(86.7)	30(100.0)		
Ethnicity					<0.001^∗^
Esan	316(91.9)	28(8.1)	344(100.0)	† = 25.273	
Beni	5(55.6)	4(44.4)	9(100.0)		
Others^∗∗^	2(28.6)	5(71.4)	7(100.0)		
Duration of marriage					0.008^∗^
10-25	300(98.7)	4(1.3)	304(100.0)	† = 11.365	
26-41	11(91.7)	1(8.3)	12(100.0)		
>42	1(50.0)	1(50.0)	2(100.0)		

Tertiary (diploma (4.2%), nursing (5.0%), polytechnic (15.5%), BSc (38.5%), MSc (10.8%), PhD (3.1%)). ^∗^Others (vocational studies (4.0%), never attended school (8.1%)). ^∗∗^Others (Igbo (0.5%), Auchi (0.5%), Hausa (0.14%), Etsako (0.4%), Yoruba (0.36%)). ^∗^Statistically significant. *χ*^2^: chi-square test; †: Fisher's exact test.

**Table 6:** Sociodemographic factors associated with parental attitude towards contraceptive use among unmarried adolescents.

Sociodemographic variables	Parental attitude towards contraceptives			Test statistics	*p* value
	Positive attitude *n*(%)	Negative attitude *n*(%)	Total *n*(%)		
Gender					
Male	24(30.8)	54(69.2)	78(100.0)	*χ* ^2^ = 2.369	0.124
Female	63(22.3)	219(77.7)	282(100.0)		
Age of respondent					
25-40	77(24.6)	236(75.4)	313(100.0)	† = 0.257	0.951
41-56	9(32.5)	31(77.5)	40(100.0)		
≥57	1(4.3)	6(85.7)	7(100.0)		
Marital status					
Married	77(25.9)	220(74.1)	297(100.0)	† = 9.001	0.022^∗^
Unmarried/single	4(9.5)	38(90.5)	42(100.0)		
Divorced	2(15.4)	11(84.6)	13(100.0)		
Widow	4(50.0)	4(50.0)	8(100.0)		
Occupation					
Civil servant	82(24.9)	247(75.1)	329(100.0)	† = 3.229	0.193
Artisan	3(11.5)	23(88.5)	26(100.0)		
Farmer	2(40.0)	3(60.0)	5(100.0)		
Education level					
Primary	3(9.4)	29(90.6)	32(100.0)	† = 21.220	<0.001^∗^
Secondary	0(0.0)	3(100.0)	3(100.0)		
Tertiary	82(29.5)	196(70.5)	278(100.0)		
Others^∗^	2(4.3)	45(95.7)	47(100.0)		
Religion					
Christianity	82(25.9)	236(74.1)	318(100.0)	† = 6.058	0.046^∗^
Muslim	3(25.0)	9(75.0)	12(100.0)		
Traditional religion	2(6.7)	28(93.3)	30(100.0)		
Ethnicity					
Esan	83(24.1)	261(75.9)	344(100.0)	† = 0.319	0.901
Beni	2(22.2)	7(77.8)	9(100.0)		
Others^∗∗^	2(28.6)	5(71.4)	7(100.0)		
Duration of marriage					
10–25	80(26.3)	224(73.7)	304(100.0)	† = 0.376	1.000
26–41	3(25.0)	9(75.0)	12(100.0)		
≥42	0(0.0)	2(100.0)	2(100.0)		

Tertiary (diploma (4.2%), nursing (5.0%), polytechnic (15.5%), BSc (38.5%), MSc (10.8%), PhD (3.1%)). ^∗^Others (vocational studies (4.0%), never attended school (8.1%)). ^∗∗^Others (Igbo (0.5%), Auchi (0.5%), Hausa (0.14%), Etsako (0.4%), Yoruba (0.36%)). ^∗^Statistically significant. *χ*^2^: chi-square test; †: Fisher's exact test.

**Table 7:** Unmarried adolescents' sociodemographic factors associated with parental willingness for contraceptive usage.

Sociodemographic variables	Parental willingness to encourage contraceptive usage				Test statistics	*p* value
	Yes *n*(%)	No *n*(%)	Maybe *n*(%)	Total *n*(%)		
Gender						
Male	27(34.6)	45(57.7)	6(7.7)	78(100.0)	*χ* ^2^ = 0.377	0.831
Female	87(30.9)	171(60.6)	23(7.3)	282(100.0)		
Age of respondent						
25-40	102(32.6)	188(60.1)	27(7.5)	313(100.0)	† = 1.852	0.748
41-56	10(25.0)	28(70.0)	2(5.0)	40(100.0)		
≥57	2(28.6)	5(71.4)	0(0.0)	7(100.0)		
Marital status						
Married	100(33.7)	177(59.6)	25(8.4)	297(100.0)	† = 9.206	0.123
Unmarried/single	10(9.5)	37(88.1)	1(2.4)	42(100.0)		
Divorced	3(23.1)	7(53.4)	3(23.1)	13(100.0)		
Widow	1(12.5)	7(87.5)	0(0.0)	8(100.0)		
Occupation						
Civil servant	102(31.0)	198(60.2)	29(8.8)	329(100.0)	† = 3.924	0.374
Artisan	9(34.6)	17(65.4)	0(0.0)	26(100.0)		
Farmer	3(60.0)	2(40.0)	0(0.0)	5(100.0)		
Education qualification						
Primary	6(18.7)	26(81.3)	0(0.0)	32(100.0)	† = 19.905	0.001^∗^
Secondary	1(33.3)	0(0.0)	2(66.7)	3(100.0)		
Tertiary	85(30.7)	167(60.3)	25(9.0)	277(100.0)		
Others^∗^	22(46.8)	23(48.9)	2(4.3)	47(100.0)		
Religion						
Christianity	103(32.4)	186(58.5)	28(8.8)	318(100.0)	† = 4.783	0.278
Muslim	4(33.3)	7(58.3)	1(8.3)	12(100.0)		
Traditional religion	7(23.3)	23(76.7)	0(0.0)	30(100.0)		
Ethnicity						
Esan	110(32.0)	206(59.9)	28(8.1)	344(100.0)	† = 1.647	0.791
Beni	2(22.2)	7(77.8)	0(0.0)	9(100.0)		
Others^∗∗^	2(28.6)	4(57.1)	1(14.3)	7(100.0)		
Duration of marriage						
10-25	99(32.6)	174(57.2)	28(9.2)	304(100.0)	† = 2.039	0.731
26-41	5(41.7)	7(58.3)	0(0.0)	12(100.0)		
≥42	0(0.0)	2(100.0)	0(0.0)	2(100.0)		

Tertiary (diploma (4.2%), nursing (5.0%), polytechnic (15.5%), BSc (38.5%), MSc (10.8%), PhD (3.1%)). ^∗^Others (vocational studies (4.0%), never attended school (8.1%)). ^∗∗^Others (Igbo (0.5%), Auchi (0.5%), Hausa (0.14%), Etsako (0.4%), Yoruba (0.36%)). ^∗^Statistically significant. *χ*^2^: chi-square test; †: Fisher's exact test.

## Data Availability

The datasets are available upon request from the corresponding author.
